# *Spirulina maxima* Decreases Endothelial Damage and Oxidative Stress Indicators in Patients with Systemic Arterial Hypertension: Results from Exploratory Controlled Clinical Trial

**DOI:** 10.3390/md16120496

**Published:** 2018-12-08

**Authors:** Jesús Martínez-Sámano, Adriana Torres-Montes de Oca, Oscar Ivan Luqueño-Bocardo, Patricia V. Torres-Durán, Marco A. Juárez-Oropeza

**Affiliations:** Departamento de Bioquímica, Facultad de Medicina, Universidad Nacional Autónoma de México, Ciudad de México 04510, Mexico; rsamano13@gmail.com (J.M.-S.); adrianatorres.bioquimica@gmail.com (A.T.-M.d.O.); luqueno@bq.unam.mx (O.I.L.-B.); pavitodu@yahoo.com.mx (P.V.T.-D.)

**Keywords:** *Arthrospira maxima*, antioxidant, cardiovascular, nutraceutical, systolic blood pressure

## Abstract

(1) Background: *Spirulina* (*Arthrospira*) *maxima* has shown beneficial effects such as being anti-dyslipidemic, antiviral, antioxidant and antihypertensive. However, there are few and limited clinical studies. (2) Methods: a prospective, randomized, parallel pilot study of 4.5 g administration of *Spirulina* *maxima* or placebo for 12 weeks in 16 patients with systemic arterial hypertension (SAH) undergoing treatment with angiotensin-converting enzyme (ACE) inhibitors was performed to assess the effects on endothelial damage and oxidative stress indicators. The blood levels of sICAM-1, sVCAM-1, endothelin-1, and sE-selectin were quantified; the activities of catalase, superoxide dismutase, glutathione peroxidase, glutathione reductase and concentrations of reduced glutathione, oxidized glutathione, and thiobarbituric acid reactive substances, were also quantified before and after the treatment period. (3) Results: There were statistically significant (*p* < 0.05) decreases in systolic blood pressure, sVCAM-1, sE-selectin and endothelin-1 levels, and increases in glutathione peroxidase activity and oxidized glutathione levels. (4) Conclusion: The effects found in the present study agree with antihypertensive and antioxidant effects previously reported for *Spirulina maxima*. However, this is the first report about the effects on indicators of endothelial damage. More research in this field is necessary to gain an insight into the effects of *Spirulina* on these indicators.

## 1. Introduction

Systemic arterial hypertension (SAH) is a syndrome of multiple etiology characterized by the persistent elevation of blood pressure levels as a response to the increase in peripheral vascular resistance resulting in systemic vascular damage [1]. Cardiovascular disease represents one of the leading causes of death worldwide, and a medical and public health problem of great importance [2]. In Mexico, the prevalence of hypertension is 25.5% among the population that is between 20 and 60 years, accounting for 15 million hypertensive patients [3]. SAH is a disease with a complex phenotype with multiple environmental and genetic risk factors, as well as environment-genotype interactions. The risk factors associated with the presence of hypertension are age [4], weight [5], genotype [6], gender and race [7]. SAH pathophysiology is also complex, and some hypotheses explain the clinical findings in patients with hypertension; for example, increase in the activity of the sympathetic nervous system [8,9], salt sensitivity [10,11], increased arterial tone and vascular remodeling, arterial stiffness [12], and renin-angiotensin-aldosterone system overactivation [13,14]. 

The vascular tone depends on equilibrium of the action of vasoconstrictor and vasodilator systems and the capacity of the vascular smooth muscle response. In normotensive subjects, the vasodilator system predominates, whereas in subjects with hypertension it is the vasoconstrictor system [15]. High and sustained blood pressure levels result in endothelial activation, dysfunction and damage [15]. It has also been described that high blood pressure correlates with endothelin-1 concentrations, reflecting the modulatory pathway of blood pressure [16]. Furthermore, intercellular adhesion molecule-1 (sICAM-1), vascular cell adhesion molecule-1 (sVCAM-1) and E-selectin in their soluble forms have been proposed as endothelial damage and inflammation markers [17].

To prevent the progression of the disease or acute and chronic complications, maintaining an adequate quality of life and reducing mortality are treatment goals for SAH. In this way, angiotensin-converting enzyme (ACE) inhibitors have shown that they have effects on the two pathophysiological mechanisms. Enalapril, zofenopril and perindopril have demonstrated their effects decreasing the oxidative stress indicator molecules [18,19], while drugs such as trandolapril, ramipril and quinapril decrease the endothelial activation and damage molecule levels [20,21].

In hypertension, an increase in reactive oxygen species (ROS) due to a decrease in the bioavailability of endothelial nitric oxide synthase with subsequent decoupling in the production of nitric oxide (NO) has been observed as well as its interaction with other molecules generating radical peroxynitrite, which has been implicated in endothelial damage [22]. The lower availability of NO and the increase in ROS are present in patients with systemic arterial hypertension. The low availability of NO induced by the pro-oxidant state is also potentiated by the effects of angiotensin II and promoted by the activation of nicotinamide adenine dinucleotide phosphate reduced (NADPH) oxidase [23].

*Spirulina maxima* (SM) has been recognized as GRAS (Generally Recognized as Safe) food by the Food and Drug Administration (FDA) and it has been shown to have several biological effects, which have been demonstrated in some clinical investigations. Thus, there are clinical studies carried out in HIV-1 seropositive subjects [24,25], with alterations in lipid metabolism [26], chronic hepatitis C virus infection [27], allergic rhinitis [28], heavy metal poisoning [29], and hypertension; but the effect of the administration of SM on sVCAM-1, sICAM-1, sE-selectin and endothelin-1 or on the indicators of oxidative stress in hypertensive patients under pharmacological control has not been evaluated. The aim of this study is to assess the effects of SM on blood pressure, antioxidant status, and endothelial damage indicators in patients with mild systemic arterial hypertension under ACE inhibitors treatment.

## 2. Results

### 2.1. Pilot Clinical Trial

**Figure 1:**
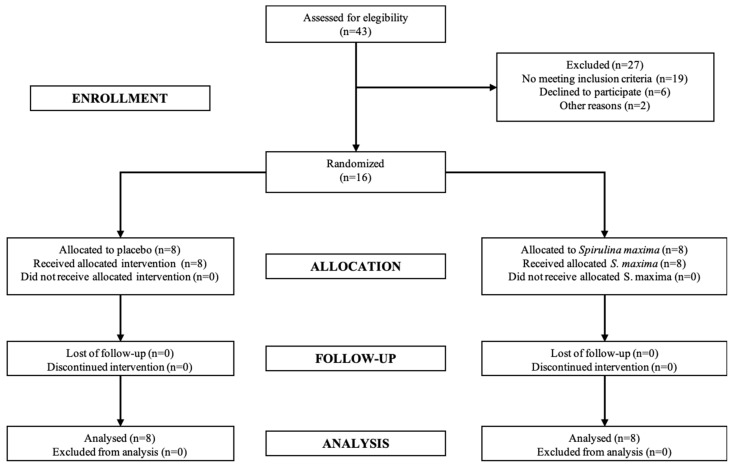
Diagram process for patients through clinical trial.

A total of 16 patients were included in the present study. The CONSORT (Consolidated Standards for Reporting Trials) diagram is shown in Figure 1. The patients fully complied with the clinical protocol procedures. Patient demographic data treated with SM or placebo did not show statistical differences (*p* > 0.05) (Table 1). There was no significant difference between the SM and placebo groups in outcome variables at baseline.

Placebo group: 7 women and one man; for SM group, 6 women and two men. BMI, Body Mass Index; HR, Heart Rate; RR, Respiratory Rate; SBP, Systolic Blood Pressure; DBP, Diastolic Blood Pressure; SAH, Systemic Arterial Hypertension; CHAL (Calidad de vida en Hipertensión ArteriaL), Quality of life questionnaire in hypertension. No statistical changes were found in both groups (*p* > 0.05).

### 2.2. Blood Pressure Levels

**Figure 2:**
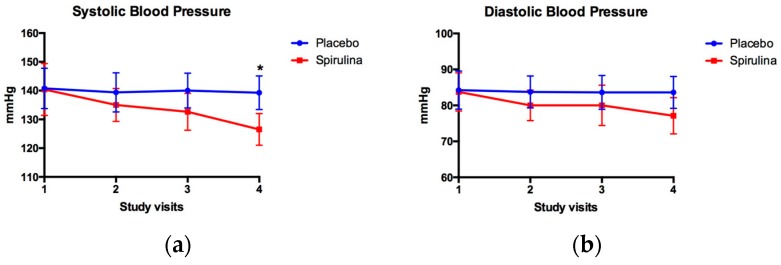
Effect of *Spirulina maxima* (SM) or placebo consumption on systolic (**a**) and diastolic blood pressure (**b**) in patients with systemic arterial hypertension. Values are presented as the mean ± standard deviation for SM (*n* = 8) or placebo (*n* = 8) groups during the follow-up period. * Statistically significant differences were observed in systolic blood pressure at the end of the 12-week period (*p* < 0.05).

Figure 2 shows the behavior of the systolic and diastolic blood pressure during 4 visits (12 weeks) in the groups treated with SM or placebo; in the case of systolic blood pressure, changes that were statistically significant were observed only in visit 4 (140.00 ± 6.05 vs. 126.50 ± 5.53 mm Hg, for placebo and *Spirulina* groups, respectively). Regarding the diastolic blood pressure, no statistically significant changes were observed throughout the treatment.

### 2.3. Endothelial Damage Indicators

For sVCAM-1 after the 12-week treatment period, a significantly lower value (*p* = 0.002) was observed in the SM group when compared with that of the placebo group. With respect to the levels of sICAM-1, no significant changes were observed between groups (*p* > 0.05) after the 12-week follow-up period. Regarding E-selectin levels at the end of the treatment, SM group showed a significantly lower value (*p* = 0.007) with respect to that of the placebo group. Finally, endothelin-1 showed a significantly lower value (*p* < 0.0002) in the group treated with SM when compared to that of the placebo group (Table 2).

### 2.4. Antioxidant Status

Table 3 shows the effect of treatment with SM or placebo on the indicators of oxidative stress in patients with systemic arterial hypertension treated with ACE inhibitors after a period of 12 weeks. CAT (Catalase), SOD (Superoxide Dismutase), GR (Glutathione Reductase) and GPx (Glutathione Peroxidase) activities were not significantly different before treatment in either group: however, after intervention, activity was higher and more statistically significant in the SM group than that in placebo group (*p* = 0.016, 0.023 and 0.0002 respectively). GR activity was lower in the placebo group after intervention (*p* = 0.0156), and GPx activity was increased in SM group after treatment (*p* = 0.0234). GSSG (Oxidized Glutathione) concentrations were higher and statistically significant in SM group compared to placebo group before intervention (*p* = 0.041), and after treatment period (*p* = 0.0002); furthermore, after intervention in the SM group, GSSG levels increased (*p* = 0.0156). No significant differences were found in GSH (Reduced Glutathione) and TBARS (Thiobarbituric Acid Reactive Substances) concentration (*p* > 0.05).

### 2.5. Quality of Life

No statistically different changes were observed in the CHAL questionnaire before starting treatment with placebo or SM (*p* > 0.05) or at the end of the intervention period for 12 weeks. The scores for the end of treatment were 24.13 ± 4.72 for the group treated with placebo and 18.75 ± 1.71 for the group treated with SM.

### 2.6. Safety

During the treatment period, a total of 29 non-serious adverse events were presented in the participating patients. In the placebo group, 7 patients presented 13 adverse events (headache, abdominal pain, diarrhea and nausea), and in the group treated with SM all patients presented at least one adverse event, accounting for a total of 16 adverse events (headache, abdominal pain, diarrhea and dizziness). There were no serious adverse events or with severity/intensity greater than Grade 2 and the evaluation of causality did not determine a certain relationship in the cases presented.

No statistically significant changes were observed in hematology, serum chemistry, urine analysis nor electrocardiogram before and after intervention in groups that were assigned to receive placebo or SM (*p* > 0.05).

## 3. Discussion

The present study was a pilot trial and the main objective was to assess the add-on effect of the oral administration of SM on the indicators of endothelial damage, antioxidant status and metabolic parameters in patients with SAH under treatment with ACE-inhibitors drugs.

### 3.1. Blood Pressure

There are a small number of clinical studies that have evaluated the effect of SM consumption on blood pressure in patients with SAH. In the study by Torres-Durán [30], the authors concluded that after supplementation with SM, 36% of patients achieved a normal blood pressure and 50% of patients had decreased blood pressure levels, classifying them in stages I or II of the Joint National Committee on Prevention, Detection, Evaluation, and Treatment of High Blood Pressure 7 (JNC7) system. Another study [31] showed that administration of 2 g/day of SM decreased blood pressure in patients with SAH. Finally, Mickze and colleagues [32] conducted a randomized, double-blind, placebo-controlled parallel group study with the administration of 2 g/day of SM or placebo for three months to a total of 40 patients with hypertension. After the treatment period, the authors reported a statistically significant decrease in systolic blood pressure and a tendency to decrease diastolic blood pressure, without being statistically significant. These findings support the results found in the present study, which shows a tendency to decrease the systolic blood pressure figures during the study visits and a statistically significant difference at the end of the 12-week treatment period.

The anti-hypertensive effect of SM in the present study would be explained by its content of C-phycocyanin and by the formation of anti-hypertensive peptides. Phycocyanin is a pigment that induces decreases in blood pressure values through increasing the expression of endothelial nitric oxide synthase [33]. The anti-hypertensive activity substances such as the tripeptide Ile-Gln-Pro (IQP), with the capacity to inhibit the angiotensin-converting enzyme, has been reported [34,35]. In vitro and in vivo studies have shown that the IC50 for IQP is 5.77 ± 0.09 μmol/L and its antihypertensive effect has been demonstrated in spontaneously hypertensive rats with a single dose in a 24 h period. A possible mechanism in the present study would be a synergistic effect between phycocyanin and the antihypertensive peptides derived from SM, which would result in a decrease in the blood pressure reported.

### 3.2. Endothelial Damage Markers

The high and sustained figures of blood pressure result in the release of mechanisms that increase vascular remodeling and endothelial activation [15]. The activation of the endothelium results in the generation of an intracellular signaling cascade that results in the expression of cell adhesion molecules and their release in soluble forms [36]. Several clinical studies in hypertensive patients have shown that elevated blood pressure results in an increase in the soluble forms of cell adhesion molecules [37,38,39,40,41,42,43]. The values reported in the present study, with the exception of E-selectin, agree with those reported in several clinical studies [37,38,39,40,41,42,43]. The changes in the endothelial damage indicators could be explained through: (1) the decrease of oxidative stimuli for the endothelium, mediated by the antioxidant compounds contained in *Spirulina* with the consequent decrease in the expression of cell adhesion molecules; (2) increase in NO concentrations mediated by the increase in endothelial nitric oxide synthase gene expression and, therefore, a decrease in vascular wall resistance with a decrease in mechanical stress to the blood vessel; and, (3) inhibition of the angiotensin-converting enzyme by the bioactive peptides, which would result in a decrease in the ligand of the AT-II (Angiotensin-II) receptor, with the consequent decrease in intracellular signaling cascades related to cell damage and, in addition, decrease in the mechanical stress induced by the angiotensin.

### 3.3. Antioxidant Status

SM has been shown to have antioxidant properties in both in vivo and in vitro studies [44,45,46,47,48,49,50,51,52,53,54,55]. In addition, it has also been shown to have an antioxidant effect in several clinical studies: for example, in patients with human immunodeficiency virus (HIV) infection, the administration of SM increases the antioxidant capacity in plasma [25]; in older adults it increases the antioxidant capacity [56]; and increases the ergogenic and antioxidant capacity in athletic subjects [57]. However, there are no publications that indicate the effect of SM on the antioxidant status of patients with SAH. The findings in the present study are consistent with those in previously established reports of an increase in antioxidant capacity when *Spirulina* is administered [25,57]. Likewise, the concentrations of the GSSG at the end of the treatment period in the group SM suggests an increase in the activity of glutathione peroxidase; this contribution of GSH in patients treated with SM could also explain the decrease in the activity of glutathione reductase, a fact that is described in the context of glutathione metabolism [58]. GSH/GSSG ratio was increased in SM group, indicating a higher content in total glutathione, as well as an increase of GPx activity, suggesting a higher bioavailability of cysteine provided by *Spirulina*.

### 3.4. Safety

The consumption of *Spirulina* has been traditional since ancient times in some regions of the world, with many considering it a safe food. The FDA of the United States accepts it as a safe supplement/food. A review of the scientific literature and of the various clinical studies indicates the lack of information on the safety profile of SM; in 30 clinical studies conducted from the year of 1987 until 2016 there is no information on adverse events or adverse reactions to *Spirulina*. In the present study, adverse events did not have a causal relationship with the administration of SM.

The work was exploratory since there are no reports in the medical literature evaluating the effect of the administration of SM on indicators of activation and/or endothelial damage. The results obtained will allow the calculation of sample size, based on the different variables determined, to carry out a larger clinical study. For future investigations it will be appropriate to perform an endothelial function test by doppler ultrasound assay to quantify the endothelium response to *Spirulina* administration.

## 4. Materials and Methods

### 4.1. Pilot Clinical Trial

An experimental prospective, pilot, parallel design, single blind, randomized, placebo-controlled study was conducted; the study was based on a screening evaluation, three follow-up visits and one final visit. Patients with stage 1–2 SAH, according to Official Mexican Standard, NOM-030-SSA2-1999 “For prevention, treatment, and control of arterial hypertension”, under stable treatment with ACE inhibitors (captopril, enalapril, lisinopril) were included; medications were given by their physician at therapeutic and recommended doses. This clinical trial was carried out in accordance with the declaration of Helsinki and was approved by Centro Especializado en Diabetes, Obesidad y Prevención de Enfermedades Cardiovasculares, S.C. (CEDOPEC) Ethics Committee No. 7-08-2014, and by the Research Committee of the Faculty of Medicine, UNAM, Mexico.

Inclusion criteria: patients who decided their voluntary participation, who understood the nature of the study and the procedures and the restrictions that involved the participation of the same, and patients older than 18 years.

Exclusion criteria: uncontrolled, stage 3 or systolic isolated hypertension, patients without pharmacological treatment, smoking history, secondary hypertension, women under pregnancy or in lactation period or who planned to become pregnant during treatment period; presence of coronary or peripheral vascular disease, diagnosed through medical history and physical examination; transaminase levels 2–3 times higher than upper normal limit, creatinine levels up to 1.5 mg/dL in screening visit.

The procedures were performed in screening, follow-up and final visits. The screening visit was carried out two weeks prior to patients’ randomization, informed consent was obtained, also demographical data, medical history, physical examination, blood pressure measurement, vital signs, electrocardiogram, and safety labs (hematology, serum chemistry, and urine analysis). If patients were eligible, they were randomized in visit 1.

The following procedures were performed in visit 1: review for inclusion and exclusion criteria, concomitant drugs review, questionnaire for quality of life in hypertension, physical examination, vital signs, blood pressure measurement and blood sampling for baseline determination of endothelial damage indicators and oxidative stress status. Additionally, in this visit SM or placebo were dispensed according with the randomization list, in sufficient quantity for patients’ consumption at the established dose until the following visit. The follow up visits 2 and 3 were performed in weeks 4 and 8 after randomization; in these visits a review of concomitant medication, adverse events and physical examination, vital signs and blood pressure measurement was performed. The final visit was performed in week 12, and the following procedures were performed: physical examination, review for adverse events, questionnaire for quality of life in hypertension, safety labs (hematology, serum chemistry, urine analysis), blood sampling for measuring endothelial damage indicators and antioxidant status, as well as an electrocardiogram. Once these procedures were completed, the clinical study was terminated for each patient who had complied with all the mentioned activities.

#### 4.1.1. Outcome Measures

The outcome measures were blood pressure levels, changes in endothelial damage indicators, antioxidant status and quality of life. The quality of life of patients with hypertension was assessed through the application of CHAL questionnaire, developed and modified in Spain by Roca-Cusachs [59]. The questionnaire assesses different aspects of the disease as well as patients’ daily life, which is affected by the disease and constant medication; it has a Cronbach’s alpha reliability index of 0.89 to 0.96. This instrument contains two dimensions: mood and somatic manifestations, which explore dimensions of hypertensive patients’ quality of life. In this sense, the CHAL questionnaire has been applied to the Mexican population [60], indicating its usefulness in this population. Blood pressure measurement was performed by a physician, following the recommendations of NOM-030-SSA2-2009, using a calibrated digital blood pressure monitor (Citizen, model CH-308B).

#### 4.1.2. Safety

Safety and tolerability of SM or placebo was carried out through the presence and severity of adverse events, in accordance with the provisions of the Official Mexican Standard, NOM-220-SSA1-2016 “Installation and operation of Pharmacovigilance. Safety labs (hematology, serum chemistry and urine analysis) and electrocardiogram performed in the screening visit and final visit by certified laboratory and interpreted by specialist physicians, according to their standard operating procedures.

### 4.2. Spirulina Maxima and Placebo Treatment

Patients were assigned to one of two treatments: SM or placebo. SM was at a dose of 4.5 g per day over a period of 12 weeks. The SM and placebo were provided by “Alimentos Esenciales para la Humanidad” (Mexico City, Mexico) in sealed bags, labeled with the patients’ number and the amount required between each study visit. The placebo was identical to SM in its organoleptic characteristics. In addition, a follow-up diary was provided for the ambulatory administration of SM or placebo, where the adverse events during treatment were recorded and the participants registered the antihypertensive treatment indicated by their treating physician. This was done in order to verify the adherence to treatment and consumption of SM or placebo.

### 4.3. Antioxidant Status Measures

After blood sampling in weeks 0 and 12, the samples were centrifuged at 4500 rpm to obtain the blood plasma, which was transferred to two polypropylene cryotubes duly labeled and stored at −78 °C under controlled conditions in ultrafreezer (Thermo Fisher Scientific, Whaltham, MA, USA) until the moment of their analyses. The sample analyses were performed in a period of 30 days after blood sampling. All the reactive were purchased from Sigma-Aldrich Química, S. de RL. de CV (Mexico City, Mexico). All spectrophotometric measurements were performed using a Genesys-10 uv spectrophotometer (Thermo Electron Corporation, Whaltham, MA, USA). Biochemical measurements were performed in duplicate with a coefficient of variation <0.05.

#### 4.3.1. Catalase

The activity of CAT was performed by the method proposed by Aebi [61], which is based on the reduction of hydrogen peroxide, through the decrease in absorbance at 240 nm. The result of the enzyme activity is expressed as catalytic units on milliliter of plasma (kat/mL).

#### 4.3.2. Superoxide Dismutase

Total activity of the SOD enzyme was based on the experiments carried out by Kono [62]. The method is based on the ability of superoxide dismutase to inhibit the reduction of nitroblue tetrazolium (NBT). The results were expressed in units/mL of plasma, where one unit is the amount of enzyme that causes the maximum inhibition of NBT by photooxidation of hydroxylamine hydrochloride. Spectrophotometric reading was performed at 560 nm.

#### 4.3.3. Glutathione Reductase

The activity of GR was based on the consumption of NADPH. The results were expressed as moles of NADPH/mL plasma per minute. The disappearance of NADPH was recorded spectrophotometrically at a wavelength of 340 nm [63].

#### 4.3.4. Glutathione Peroxidase

Glutathione peroxidase activity was based on the method developed by Paglia and Valentine where the moles of oxidized NADPH per minute/mL of plasma are determined. This technique is based on the ability of peroxidase to reduce organic peroxides through the oxidation of two GSH molecules. The oxidation of NADPH was monitored at 340 nm [64].

#### 4.3.5. Reduced and Oxidized Glutathione

Measurement of GSH was based on the Ellman reaction, a plasma sample reacted with dinitrobenzene in the presence of phosphate buffer. A standard curve was made with known concentrations of glutathione, the results being expressed as mg of GSH/mL of plasma. The spectrophotometric reading was performed at 412 nm. For the case of GSSG, the samples were treated with 4-vinylpyridine, to precipitate the reduced glutathione, leaving only the oxidized glutathione as substrate for the assay.

#### 4.3.6. Thiobarbituric Acid Reactive Substances

The final products of the peroxidation were evaluated as TBARS as described by Torres-Durán et al. [65]. A standard curve was made with tetraethoxypropane and the results were obtained extrapolating from the standard curve, expressing them as ng/mL plasma.

### 4.4. Endothelial Damage Indicators

Measurements of the soluble forms of sICAM-1, sVCAM-1 and sE-selectin, as well as levels of endothelin-1, were carried out by commercial ELISA kits (Millipore, Burlington, MA, USA) following the manufacturer’s instructions. All ELISA determinations were performed using an EPOCH^TM^ microplate reader (Biotek, Winooski, VT, USA).

### 4.5. Statistical Analyses

The statistical analysis was performed with SPSS software v.11; a t for unpaired groups and Mann-Whitney tests were carried out considering a *p* < 0.05 as statistically significant.

## 5. Conclusions

The results obtained demonstrate that the administration of SM has an add-on effect in the treatment of arterial hypertension; it has an effect on the decrease of indicators of endothelial activation damage (sVCAM-1, E-selectin and endothelin-1); as well as increasing antioxidant defenses (increased activity of SOD, GPx, and GSSG concentrations) supporting previous research on the antioxidant properties of *Spirulina*. This is the first clinical study that demonstrates the effect of cyanobacteria on indicators of endothelial damage, which makes it necessary to perform clinical studies of greater proportions to have sufficient statistical power to support the findings of this research.

## Figures and Tables

**Table 1:** Baseline characteristics of patients enrolled in the study.

Baseline Parameter	Placebo Group (*n* = 8)	SM Group (*n* = 8)
Age (years)	51.80 ± 9.44	57.00 ± 8.66
Weight (kg)	77.20 ± 18.89	79.36 ± 29.40
Height (m)	1.51 ± 0.10	1.56 ± 0.13
BMI (kg/m^2^)	34.19 ± 10.69	31.56 ± 7.11
HR (bpm)	71.20 ± 9.23	82.00 ± 7.34
RR (bpm)	19.40 ± 0.54	19.00 ± 1.00
Temperature (°C)	36.20 ± 0.27	36. 26 ± 0.35
SBP (mm Hg)	140.75 ± 7.03	140.38 ± 9.04
DBP (mm Hg)	84.25 ± 5.28	83.75 ± 5.31
SAH evolution (years)	4.20 ± 3.11	4.40 ± 3.05
CHAL (points)	25.20 ± 15.48	28.80 ± 11.56

**Table 2:** Effects of SM or placebo in endothelial damage indicators in patients with systemic arterial hypertension (SAH). Values are shown as mean of 8 subjects per treatment group (SM or placebo) ± standard deviation. *, compared vs. placebo group in basal conditions; ** compared vs, placebo group in post-treatment. ^+^ compared vs. SM group in before treatment. VCAM-1, vascular-cell adhesion molecule-1; ICAM-1, intercellular adhesion molecule-1.

Indicator	Pre-Treatment			Post-Treatment			Intra Group Comparison (*p*)
	Placebo (*n* = 8)	*Spirulina maxima* (*n* = 8)	*p*	Placebo (*n* = 8)	*Spirulina maxima* (*n* = 8)	*p*	
sVCAM-1 (ng/mL)	508.90 ± 31.40	480.80 ± 10.30 *	* =0.033	501.60 ± 22.70	458.00 ± 19.60 **^,+^	** 0.0022	^+^ 0.0391
sICAM-1 (ng/mL)	375.10 ± 96.40	348.50 ± 85.20	0.64	308.50 ± 99.10	333.30 ± 99.10	0.88	0.64
sE-Selectin (ng/mL)	13.73 ± 4.16	12.12 ± 1.52	0.77	12.40 ± 1.24	9.71 ± 2.13 **	** 0.007	^+^ 0.0234
Endothelin-1 (pg/mL)	17.45 ± 6.11	14.42 ± 2.92	0.22	21.48 ± 2.17	11.90 ± 6.47 **	** 0.0002	^+^ 0.0391

**Table 3:** Effects of SM or placebo in antioxidant status from patients with SAH. Values are shown as mean of 8 subjects per treatment group (SM or placebo) ± standard deviation. *, compared vs. placebo group in basal conditions, ** vs. placebo group after 12-week treatment period, ^+^ compared vs. placebo group before treatment, ^Δ^ compared vs. SM group before treatment. CAT, catalase; SOD, superoxide dismutase; GR, glutathione reductase; GPx, glutathione peroxidase; GSH, reduced glutathione; GSSG, oxidized glutathione; TBARS, thiobarbituric acid reactive substances.

Oxidative Stress Indicator	Pre-Treatment			Post-Treatment			Intra Group Comparison (*p*)
	Placebo (*n* = 8)	*Spirulina maxima* (*n* = 8)	*p*	Placebo (*n* = 8)	*Spirulina maxima* (*n* = 8)	*p*	
CAT (*k*/mL)	3.35 ± 1.09	3.43 ± 1.15	0.800	3.35 ± 1.11	3.76 ± 1.23 **	** 0.016	0.054
SOD (U/mL)	77.63 ± 1.47	77.67 ± 1.87	0.980	76.63 ± 0.43	82.40 ± 1.32 **	** 0.0023	^Δ^ 0.009
GR (µmol NADPH/min)	61.45 ± 18.27	52.17 ± 28.32	0.480	41.49 ± 3.36 ^+^	51.93 ± 12.44 **	0.053	^+^ 0.0156
GPx (µmol NADPH/min)	354.70 ± 79.86	355.71 ± 42.26	0.490	322.43 ± 36.36	404.27 ± 25.89 **^,^^Δ^	** 0.0002	^Δ^ 0.0234
GSH (mg/mL)	23.51 ± 5.08	23.35 ± 6.13	0.840	22.82 ± 2.32	26.94 ± 4.01	0.083	0.312
GSSG (mg/mL)	19.96 ± 3.32	26.76 ± 5.93 *	* 0.041	20.01 ± 1.58	37.88 ± 7.54 **^,^^Δ^	** 0.0002	^Δ^ 0.0156
TBARS (µg MDA/mL)	5.74 ± 3.79	4.47 ± 2.68	0.570	3.89 ± 1.59	3.90 ± 1.16	0.999	0.740
